# Editorial: Cortical Maps: Data and Models

**DOI:** 10.3389/fninf.2022.954042

**Published:** 2022-06-17

**Authors:** Nicholas V. Swindale, Geoffrey J. Goodhill

**Affiliations:** ^1^Department of Ophthalmology and Visual Sciences, University of British Columbia, Vancouver, BC, Canada; ^2^Departments of Developmental Biology and Neuroscience, Washington University in St. Louis, St. Louis, MO, United States; ^3^Queensland Brain Institute and School of Mathematics and Physics, The University of Queensland, St Lucia, QLD, Australia

**Keywords:** cortical maps, topographic maps, somatotopic maps, tonotopic maps, columns, orientation maps

In our original solicitation for papers on the topic of cortical maps, we posed a number of currently unanswered questions. How widespread are maps in the cortex? Are there, for example, ordered representations of speech-related properties in Broca's area? Are there maps of some kind in frontal cortical areas? To what extent are map details dependent on innate, genetically programmed mechanisms, or on patterns of neural activity resulting from sensory experiences and motor behaviors? Despite the interest they have aroused, might maps be epiphenomena with no real function (Horton and Adams, 2005)? These issues, among others, are addressed by the articles in this collection. They range from reviews (Grossberg, Ibbotson and Jung, and Sereno), modeling studies (Grossberg, Tanaka et al.), descriptive advances in the classification of pathways and areas (Johnson et al., Wojtasik et al., and Choi et al.) and experimental and modeling studies of critical period plasticity (Tanaka et al., Thomas et al.).

Sereno et al. address the issue of the ubiquity of maps by showing that the most basic kinds of topographic map: retinotopic, somatotopic and tonotopic, cover half the cortical surface in humans. The authors also point out that patchy local connectivity has been found in many cortical areas, including those where the evidence for topographic maps is limited or lacking. Patchy local connections seem a very strong indicator of fine scale columnar organization of functional properties, given that connected sets of neurons are likely to share functional properties. Hence even if some types of map are absent in some species, maps and columns in general appear to be ubiquitous features of cortical organization. The article by Grossberg shows how maps and columns may enable computations based on canonical wiring rules such as Gaussian, or difference of Gaussian, interactions mediated in the space of the cortex by branching axons. Rules of this nature have the consequence of reducing the developmental demands on the axonal wiring involved—not just the lengths of the wires but in making fewer demands on genetic instructions. More importantly, Grossberg's article provides a valuable summary of almost half a century of modeling work done by himself and his colleagues. The various models unite low-level facts of cortical anatomy and physiology with cognitive operations in a way that is rarely attempted. The models are ambitious but they provide a firm foundation for future tests and development that may be exceedingly valuable.

As Grossberg's article makes clear, maps provide a context which makes studying the functions that are carried out in them much easier. The existence of maps has also made many developmental processes more accessible to study: for example the dramatic change in the widths of ocular dominance stripes demonstrated by Hubel et al. (1977) following monocular deprivation was surely much more informative than the corresponding change in the shape of the ocular dominance histogram. Maps have also highlighted and simplified the understanding of critical periods in development, since structural map plasticity is often a key indicator of these periods. Two papers in the collection deal with this problem. Thomas et al. study the consequences of auditory map plasticity induced by rearing rat pups in an environment where a continuous 7 KHz tone was audible, resulting in an increase in the area of the map devoted to that frequency. There is evidence that this kind of alteration may contribute to conditions such as tinnitus and sensitivity to loud sounds. Tanaka et al. combine modeling and experimentation to study plasticity of orientation maps. The maps have a critical period and in general the experimental and modeling data agree beautifully well.

Like many previous modelers, Tanaka et al. employ stimulation with oriented gratings to produce correlated activity in cortical neurons which then leads to the development of an orientation map. Until recently, this might have been considered an unrealistic assumption as there is good evidence that orientation selectivity and orientation maps are present at birth in monkeys (Wiesel and Hubel, 1974) as well as shortly after birth in visually deprived cats (Crair et al., 1998) and it has been presumed that visual stimulation can only occur after birth, and if the eyes are open. However it is becoming clear that environmental visual stimulation can occur *in utero* (Del Giudice, 2011) and that the human fetus, whose eyes open before birth, is responsive to visual stimuli (Reid et al., 2017). If this is true for humans, it is likely to be even more so for other mammals, who do not wear clothes and have very much thinner uterine and abdominal walls. So the assumption that vision (beyond the limited patterns provided by retinal waves) cannot be present before birth, and which has colored work on map development for decades, may be wrong.

Comparative studies of maps in different species almost always seem to be illuminating, especially since maps have been studied in only a handful of species. The article by Ibbotson and Jung reviews the factors that might determine the presence or absence of ordered orientation maps. Although phylogeny is currently the best predictor, an alternative, more directly related to visual system function, is the ratio of central and peripheral ganglion cell densities. This idea is consistent with existing data as well as more recent findings that the agouti (a large rodent) lacks maps (Ferreiro et al., 2021) and that wallabies have them (Jung, 2020).

Finally, an important aspect of work on maps involves the description of new areas and making the descriptions accessible. Three articles in the collection address this issue. Sereno et al. provide a downloadable annotation and parcellation of 115 human cortical areas with topological maps in the FreeSurfer *fsaverage* surface. Johnson et al. provide a DTI atlas for the cat available at https://ecommons.cornell.edu/handle/1813/58775.2 Four new cytoarchitectonically defined areas of human orbitofrontal cortex are described by Wojtasik et al. and are available at a number of sites given in the article.

## Author Contributions

NVS wrote the text which was revised in response to comments and suggestions by GJG. Both authors have approved the final version.

## Conflict of Interest

The authors declare that the research was conducted in the absence of any commercial or financial relationships that could be construed as a potential conflict of interest.

## Publisher's Note

All claims expressed in this article are solely those of the authors and do not necessarily represent those of their affiliated organizations, or those of the publisher, the editors and the reviewers. Any product that may be evaluated in this article, or claim that may be made by its manufacturer, is not guaranteed or endorsed by the publisher.
